# Despite Inflammation, Supplemented Essential Amino Acids May Improve Circulating Levels of Albumin and Haemoglobin in Patients after Hip Fractures

**DOI:** 10.3390/nu9060637

**Published:** 2017-06-21

**Authors:** Roberto Aquilani, Ginetto Carlo Zuccarelli, Anna Maria Condino, Michele Catani, Carla Rutili, Consiglia Del Vecchio, Pietro Pisano, Manuela Verri, Paolo Iadarola, Simona Viglio, Federica Boschi

**Affiliations:** 1 Dipartimento di Biologia e Biotecnologie Università degli Studi di Pavia, Via Ferrata, 1. I-27100 Pavia, Italy; dottore.aquilani@gmail.com (R.A.); manuela.verri@unipv.it (M.V.); 2 Istituto Geriatrico P. Redaelli -Reparti di Riabilitazione Geriatrica e di Mantenimento, Via Leopardi, 3. I-20090 Vimodrone, Milano, Italy; zuccarelli1@msn.com (G.C.Z.), m.catani@golgiredaelli.it (M.C.); c.rutili@golgiredaelli.it (C.R.); lidiadelvecchio@libero.it (C.D.V.); p.pisano@golgiredaelli.it (P.P.); 3 Dipartimento di Scienze del Farmaco, Università degli Studi di Pavia, Viale Taramelli, 14. I-27100 Pavia, Italy; annamaria.condino@unipv.it; 4 Dipartimento di Biologia e Biotecnologie Università degli Studi di Pavia, Via Ferrata, 1. I-27100 Pavia, Italy; paolo.iadarola@unipv.it; 5 Dipartimento di Medicina Molecolare, Università degli Studi di Pavia, Viale Taramelli, 3/B. I-27100 Pavia, Italy; simona.viglio@unipv.it

**Keywords:** albumin, haemoglobin, essential aminoacids, elderly hip fracture

## Abstract

Essential amino acids (EAAs) are nutritional substrates that promote body protein synthesis; thus we hypothesised that their supplementation may improve circulating albumin (Alb) and haemoglobin (Hb) in rehabilitative elderly patients following hip fractures (HF). Out of the 145 HF patients originally enrolled in our study, 112 completed the protocol. These subjects were divided into two randomised groups, each containing 56 patients. For a period of two months, one group (age 81.4 ± 8.1 years; male/female 27/29) received a placebo, and the other (age 83.1 ± 7.5 years; male/female 25/31) received 4 + 4 g/day oral EAAs. At admission, the prevalence of both hypoAlb (<3.5 g/dL) and hypoHb (<13 g/dL male, <12 g/dL female) was similar in the placebo group (64.3% hypoAlb, 66% hypoHb) and the treated group of patients (73.2% hypoAlb, 67.8% hypoHb). At discharge, however, the prevalence of hypoAlb had reduced more in EAAs than in placebo subjects (31.7% in EAAs vs. 77.8% in placebo; *p* < 0.001). There was a 34.2% reduction of anaemia in hypoHb in EAA subjects and 18.9% in placebo subjects, but the difference was not statistically significant. Oral supplementation of EAAs improves hypoAlb and, to a lesser extent, Hb in elderly rehabilitative subjects with hip fractures. Anaemia was reduced in more than one third of patients, which, despite not being statistically significant, may be clinically relevant.

## 1. Introduction

Circulating albumin (Alb) and haemoglobin (Hb) proteins are considered to be indicators of the status of general health [1] both in community and clinical settings (acute-, long-term care-, rehabilitation-environments). Low Alb in community-dwelling healthy elderly individuals is independently associated with poorer performance [2] and predicts a greater decline in functional status [2,3]. However, in a clinical setting, low Alb correlates with disease severity and mortality [1,4,5], predicts a prolonged hospital stay, and increases the complication rate and all-cause mortality [5,6,7]. In institutionalised populations, subjects with hypo-albuminemia (hypoAlb) have increased mortality, which is independent of age, sex, medication use, or protein intake [8]. In the rehabilitation ward, an increase in Alb in elderly patients with hip fractures [9] or ischemic strokes [10] predicts higher functional independence in patients.

With regard to Hb, reduced protein blood levels predispose community-dwelling elderly individuals to the frailty syndrome [11] by inducing alterations in skeletal muscle mass density and strength, which are both responsible for impaired physical performance [12], increased risk of disability [13,14], and impaired quality of life [15]. Moreover, low Hb has been documented to be an independent factor of increased mortality [16,17] in hospitalized medical and surgical patients, and the degree of anaemia is associated with short-term mortality in many studies [18].

One of the patient populations that suffers from anaemia is elderly individuals with hip fractures (HF). The prevalence of anaemia observed in elderly HF at discharge from surgical wards is very high (84%) [19] due to significant blood loss following fractures, surgery, and possible post-operative complications. For the first few days after surgery, anaemia is one of the main factors that delays patient mobilisation [20]. Early mobilisation is the best predictor of both a patient’s reduced mortality over one year after trauma and discharge from hospital [21].

Interestingly, Alb and Hb seem to be interrelated and to vary in the same way in older subjects [22]. Alb has been observed to be 1 g/L lower in anaemic individuals compared to normal subjects, and anaemia is associated with a seven-fold higher chance of hypoAlb [22].

Based on all of these studies, we believe that physicians should try to decrease hypoAlb and/or anaemia during a patient’s hospital stay. Even though this is also true for Hb, its levels do not seem to negatively influence functional independence either in the acute rehabilitation period [23], or later on after hospital discharge [24]. We believe that Hb improvement should be pursued because anaemia is associated with several adverse outcomes such as the development of cardiovascular and renal diseases [20], death, functional dependence, dementia, and falls [25,26,27,28].

As a consequence, improving both hypoAlb and anaemia is of great importance for both individual health and the economic sustainability of the health system. However, improving these circulating proteins in patients with inflammation following trauma and surgery may be difficult, particularly when patients have inadequate protein-energy intakes [29].

In the current study, we aimed to investigate whether supplementation with essential amino acids (EAAs) could improve hypoAlb and anaemia in rehabilitative elderly subjects with HF surgery and mild hypoAlb and anaemia (i.e., not requiring Alb intravenous infusion or transfusion, testosterone, or erythropoietin use). Although a previous study found a negative effect of amino acid supplement on glucose homeostasis, inflammatory markers, and incretins after laparoscopic gastric bypass (Breitman I J Am Coll Surg 2011), we believed there was a strong rationale for using EAAs.

Firstly, these substrates boost protein synthesis [30]. This has even been found to occur during severe inflammation such as that induced by endotoxin [31]; secondly, EAAs have been reported to increase albumin concentrations in sarcopenic patients with chronic obstructive pulmonary disease [32] and in elderly institutionalised individuals [33]; and thirdly, EAAs increase Hb concentrations in haemodialysed subjects [34].

Lastly, EAAs can reduce infection, which negatively impacts albumin and HB syntheses [35].

We therefore studied a cohort of elderly patients admitted to our Geriatric Intensive Rehabilitation Institute after surgery for HF.

## 2. Subjects and Methods

### 2.1. Population and Measures

The patients enrolled in this study were consecutively admitted between 1 December 2009 and 30 November 2010. They came straight from the Department of Orthopaedic surgery 20 ± 5 days after undergoing HF surgery. They were all clinically stable and thus received active rehabilitation therapy after surgery for pertrocanteric or sub capital HF. Patients were included independently of their serum levels of Alb because, even within the normal range of values, lower levels of the protein could put the patient at risk of mortality and disease [8]. Furthermore, Alb levels that are higher than the clinical cut-off of 3.8 g/dL are associated with a future loss of skeletal muscle mass (sarcopenia) [2].

The exclusion criteria for patients in our study were as follows: antibiotic therapy on admission, body temperature >36.8 °C, diabetes on insulin treatment, cancer or non-operated cancer, pressure ulcer(s), haematological cancer, acute or advanced chronic renal failure (serum creatinine >2 mg/dL), heart failure, or cognitive alterations (Mini Mental State Examination, <24 scores).

All subjects gave their informed consent for inclusion before they participated in the study. The study was conducted in accordance with the Declaration of Helsinki, and the protocol was approved by the Ethics Committee of the Institute (Direzione Generale/Atti/2008/FF/ R002/13.2.2008).

During the first 48 h after admission, patients underwent the following assessments:

(1) Anthropometric measurements: body weight (kg) was determined using a mechanical weight lifter; height (m) was calculated from knee height [36]; body mass index (BMI) was calculated as kg/m^2^; although 70% of patients were able to stand up, we preferred to weigh them by mechanical lifter to avoid instability when they were on the base of the weighing scale.
(2) After overnight fasting, at 7:00 a.m. blood samples were taken from peripheral veins to determine routine variables, which included the measurements of serum/blood protein concentrations (total protein-TP, Alb, prealbumin-preAlb, C-reactive protein (CRP)).

Total serum protein concentrations (normal value 6–8 g/dL) were determined using colorimetric methods (Biurete Colour, Dimention RXL Siemens, Munich, Germany). Alb was measured with capillary electrophoresis (Mini Capillary, Sebia, Cedex, France) and expressed as a percentage of TP (normal value 55.8–66.1%). Serum Alb concentrations were then obtained by multiplying the percentage of Alb by TP (normal value 3.5–5 g/dL). Hypoalbuminemia was indicated as a value <3.5 g/dL. Serum preAlb (normal range 20–40 mg/dL) was measured with an immuno-turbidimetric method (Cobas CCE Roche, Tokyo, Japan). Low preAlb was indicated by a value <20 mg/dL. Serum C-reactive protein (CRP) concentrations were determined with an immuno-turbidimetric method (Dimention RXL Siemens, Munich, Germany). CRP level >0.8 mg/dL was used as a marker of body inflammation. Blood Hb was analysed with a photometric method (Counter XE-2100, Dasit Symex Corporetion, Kobe, Japan). Hb concentrations <13 g/dL in men and <12 g/dL in women indicated the presence of anaemia [19].

### 2.2. Nutritional Intake

As described elsewhere [35], a three-day food diary was prepared for each patient by nurses who used a diet sheet to keep a record of the type and weight of cooked/uncooked food selected by patients from the hospital catering menu, both before and after their meals. Subsequently, we performed a nutritional analysis to calculate the actual calories and macro/micronutrients that the patients had ingested [35] by using the computer program Food Database DR3 (Dieta ragionata 3. Sintesi Informatica. University of Pavia, Italy). In brief, this program contains all food items and the energy concentrations of macronutrients (kcal/100 g nutrients) and, respectively, of raw and cooked foods. By entering the cooked/uncooked food item that the patient actually ingested into the database, the energy values (Ė) and macronutrients were calculated by multiplying the weight of the ingested food by its energy density and macro-micronutrient makeup.

### 2.3. Co-Morbidities

Associated disease(s) with the primary disease (HF) were analysed by the Charlson Index [37].

### 2.4. Patient Randomisation

After completing all of the above procedures, the patients were assigned a treatment according to a randomised allocation procedure. A randomisation list was generated using Statistical Analysis System SAS software; A and B were the identifiers of the blind treatment. The list was made available to both physicians (G.Z. and C.M.) and the hospital pharmacist. The physicians sequentially allocated patients to treatment A or B according to the randomised list. The first author (R.A.) who interpreted all the results was blinded to patient allocation. The experimental group (EAA group) received an oral nutritional mixture supplement, which provided 8 g of EAAs/day (Aminotrophic, Professional Dietetics, Milan, Italy; 4 g in the morning + 4 g in the afternoon, diluted in half a glass of water) for 60 days.

Each EAA package contained leucine 1250 mg, valine 625 mg, isoleucine 625 mg, lysine 650 mg, threonine 350 mg, cystine 150 mg, histidine 150 mg, phenylalanine 100 mg, methionine 50 mg, tyrosine 30 mg, and tryptophan 20 mg. We chose 8 g EAAs as this dose was found to be effective in several severe chronic diseases to improve insulin resistance [32,33,38] and serum albumin concentrations [32,33].

The calorie content of the single amino acid package was 21.9 kcal (EAA mixture) and 20.2 kcal (casein) (Table 1).

The placebo group (control group) was given a similar isocaloric, isonitrogenous (casein) product. The nurses assisted each patient during the intake of either the placebo or EAAs in order to be certain of patient compliance.

The duration of the study was 60 days from the randomisation procedure.

All the above procedures were repeated 30 days (T1) and 60 days (T2) after the protocol started (T0).

### 2.5. A Rehabilitation Protocol

The protocol aimed to restore complete functional recovery of the altered body status, the resumption of a walking pattern that was as normal as possible, and of daily life activities (DLA).

The rehabilitation protocol consisted of two sessions per day, five days per week. Each session lasted 40–50 min and included the following main steps:

- Range of motion (ROM): a passive and assisted active mobilisation of the limb that had been operated on (15 min)
- Muscle strength:
  - isotonic and isometric exercises, neuromuscular facilitation of the sural triceps muscles (three sets of 10 repetitions; 15 min)
  - isotonic exercise and against resistance of: (1) abdominal trunk muscles to contrast the anteversion of the pelvis and (2) Gluteus maximus muscle to restore leg extension movement; (3) Gluteus medius and minimus muscles to keep the pelvis static and to be able to walk without oscillation (3 sets of 10 repetitions; 15 min)
- Assisted gait training with the use of walking sticks (10 min).

### 2.6. Statistical Analysis

The sample size estimate was determined by performing an appropriate power analysis. More specifically, based on preliminary data, we planned to observe an effect size f(V) (derived from preliminary partial η^2^) between treatment groups of about 0.36. Starting from this hypothesis and assuming a type I error of 5% (α = 0.05) and a type II error of 10% (power = 0.9), the sample required consisted of 50 patients per group.

Descriptive statistics were performed for all the recorded variables, and data were summarised as mean ± standard deviation (SD). The baseline characteristics between EAA and placebo groups were compared using an independent sample Student’s *t* test or chi-square test, as appropriate. Comparisons of trends over time between EAA and placebo groups were performed by applying a repeated measure (times: baseline (T0), day 30 (T1), day 60 (T2)) analysis of variance (ANOVA), with one factor. Specific contrasts were estimated in order to assess differences between the two time points. A repeated measures ANOVA with two factors was used to test the influence on time trends of both treatment supplementation and the presence of infections. The difference in the prevalence of any developed infection between the two groups was tested by performing a chi-square test.

The previous analyses were also carried out to test the variation of the protein concentrations due to the treatment over time in female and male patients.

Linear regression analysis and Pearson’s correlation coefficient were estimated to assess the relationship between protein concentrations (as absolute values at admission and discharge and as differences between discharge and admission) and functional tests. Statistical significance was set at *p* < 0.05.

## 3. Results

**Figure 1:**
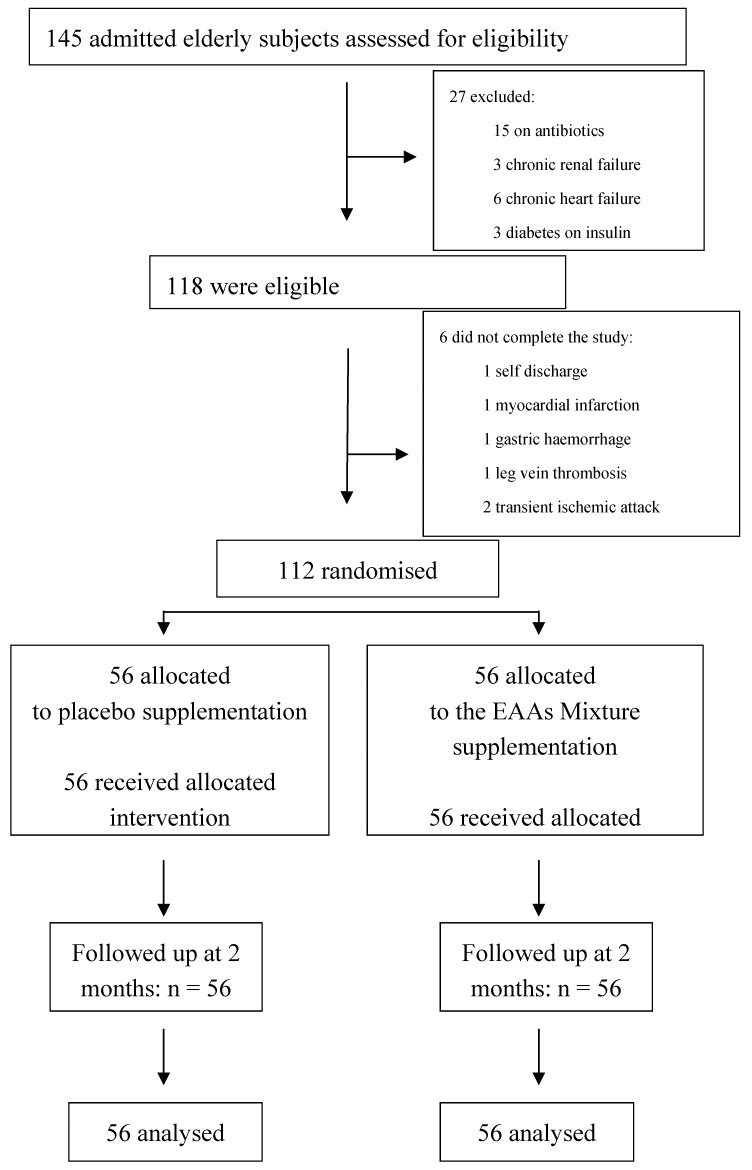
Flow diagram of a trial supplementation with Essential amino acids (EAAs) mixture vs. placebo to treat elderly patients with hip fractures. The diagram includes the number of patients analyzed for the main outcomes (effect on circulating proteins).

A total of 145 patients with hip fractures (HF) were enrolled, 118 of which met the inclusion criteria and were included in the study (Figure 1): 15 patients were excluded because of antibiotic therapy at or up to two days before admission to the Rehabilitation Institute; three for chronic renal failure, six for chronic heart failure, and 3 for diabetes on insulin treatment.

A total of 112 out of the 118 patients included in our study completed the study protocol. More specifically, six patients (two in the treated group and four in the placebo group) discontinued the study because of self-discharge (*n* = 1), myocardial infarction (*n* = 1), gastric haemorrhage (*n* = 1), leg deep vein thrombosis (*n* = 1), and transient ischemic attack (*n* = 2). Both the placebo and treated groups included 56 patients. Sixty percent of the placebo group and 65% of the EEA group were given at least one transfusion in the perioperative phase.

### 3.1. Baseline Characteristics

After randomisation, the treated and placebo groups were similar for co-morbidity (Charlson Index), demographic-anthropometric characteristics, nutritional intake, blood glucose, and urea concentrations. The placebo group was mildly overweight, and the EAA group had a normal body weight (categorisation by using the World Health Organisation WHO database). The daily calorie and protein intakes were slightly lower than recommended for both groups [39] (Table 2). The nutritional analysis conducted on the three-day diaries, showed that in both groups (a) proteins were mainly of animal origin (77.6 ± 4.8% in placebo vs. 78.9 ± 3.6% in treated group; from fresh/cured meats, fish, eggs, milk/dairy products, cheese); (b) EAA dietary intakes were similar; (c) ingested simple sugar (sugar, yoghurt/milk/fruit) comprised 17% of the daily energy intake, which was mildly higher than the recommended amount (<15%); and (d) there was an increased lipid intake with normal ingestion of saturated fats. However, the ingestion of ω3 fatty acids was lower than the recommended amount. A status of systemic body inflammation, indicated by CRP levels (Table 3), was present in and similar for the two patient populations.

**Figure 2:**
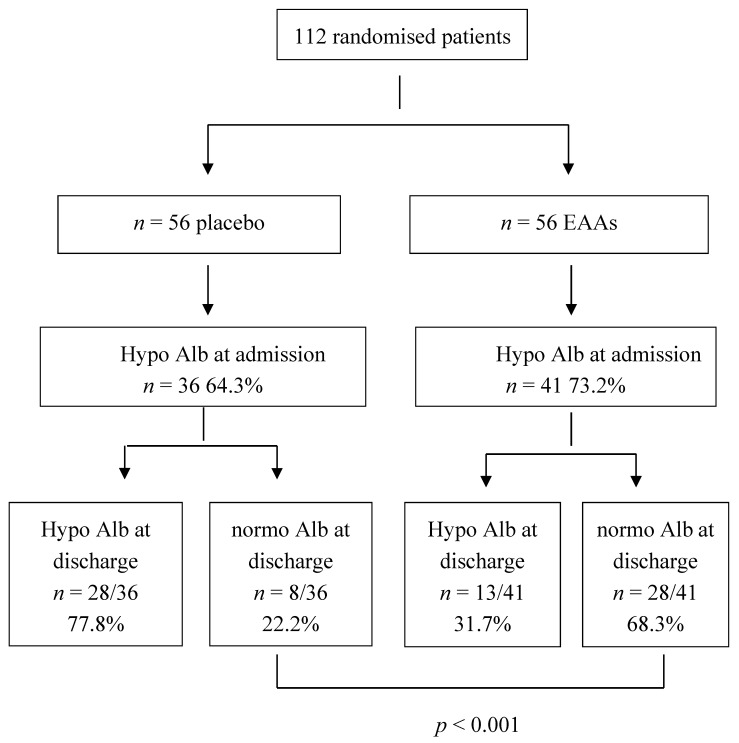
Flow diagram showing the percentage of admitted patients with hypoalbuminemia (Alb < 3.5 g/dL) who did not improve or improved albumin (Alb) during the Rehab period.

As regards serum proteins (Table 3), Alb was lower than the normal range of values in the entire population. The prevalence of hypoAlb (Figure 2) was similar between treated and placebo subjects (64.3% in placebo and 73.2% in treated patients; ns). The prevalence of anaemia in the entire study population was 67% and was distributed similarly between the two groups (66% in placebo and 67.8% in treated patients; ns).

The results showed that Alb significantly correlated with Hb (*r* = +0.397; *p* < 0.001).

### 3.2. Variable Changes during the Rehabilitation Phase

The results showed significant differences (*p* < 0.05) in the overtime trends of Alb between the two patient populations. In placebo patients, the serum Alb concentrations remained virtually unchanged (Table 3), whereas in treated subjects, the serum Alb concentrations progressively increased over time (Table 3). However, no significant sex-based differences emerged from the model (*p* = 0.74). The normalisation of Alb levels (≥3.5 g/dL) occurred in 68.3% of the EAA group and in 22.2% of the placebo group (*p* < 0.001) (Figure 2). The average Alb improvements were +0.46 ± 0.43 g/dL in EAAs and +0.22 ± 0.19 in placebo patients.

At discharge, a status of hypoAlb was still present in 77.8% of placebo patients and in 31.7% of EAAs.

As regards Hb (Table 3), the changes in this protein reflected those of the Alb. Indeed, Hb changed very little in the placebo group, whereas it progressively increased in the treated subjects. This difference was significant (*p* = 0.008). More specifically, in the EAA group, the time course of blood Hb content was different between baseline (T0) and T2 (*p* = 0.003), and between T1 and T2 (*p* = 0.002) and tended to be significant between T0 and T1 (*p* = 0.08). As with Alb concentration, no significant differences of Hb concentration between male and female subjects emerged from the model (*p* = 0.5).

**Figure 3:**
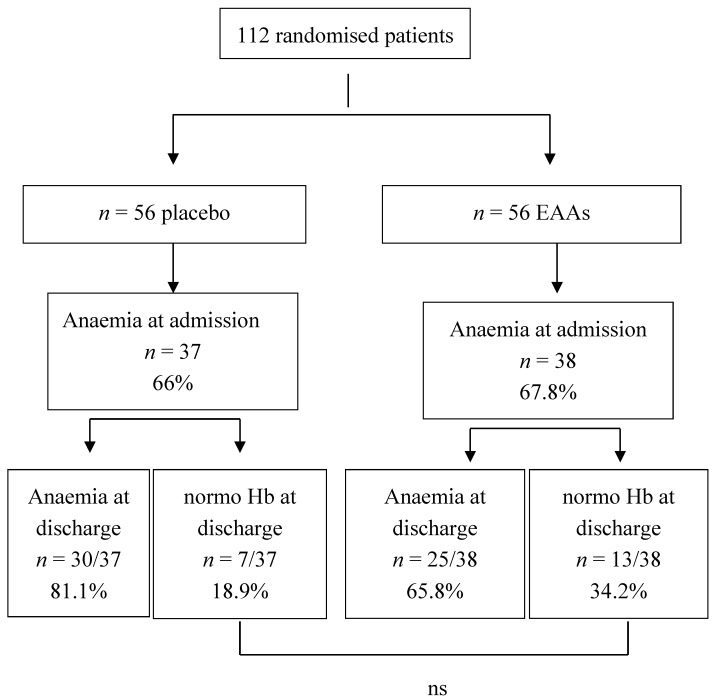
Flow diagram showing the percentage of admitted patients with anaemia (Hb < 13 g/dL male; <12 g/dL female) who did not improve or improved haemoglobin (Hb) during Rehab period.

Figure 3 shows that improvements in Hb occurred in 18.9% of placebo subjects and in 34.2% of EAAs. In absolute values, the rates of improved Hb were higher in EAAs than in placebo subjects (+0.75 ± 0.34 g/dL vs. +0.25 ± 0.31 g/dL) (*p* < 0.01).

At discharge, more than 80% of placebo anaemic subjects and more than 65% of treated subjects were still anaemic (Figure 3).

At discharge, Alb and Hb showed a significant correlation (*r* = +0.5, *p* < 0.001).

There were no significant changes of CRP over time either in the placebo or in the EAA group to indicate the persistence of systemic inflammation. No significant changes were observed in either male or female patients (*p* = 0.48). Infections (namely of urinary and lower airway tracts) were higher in placebo (80%) than in treated patients (55%) (*p* < 0.02). Infection did not modify the EAA effects on Alb time courses (*p* = 0.46) or Hb (interaction treatment *p* = 0.73).

After two months of rehabilitation, there was no significant change between baseline body weight or daily nutritional intakes in either patient group or in pre-Alb concentration levels (or in male or female patients) (*p* = 0.8)

To summarise, supplemented EAAs were associated with improvements in Alb and, to a lesser extent, Hb. At about 80 days from the index event, both placebo and EAA subjects were discharged with persistent systemic inflammation.

## 4. Discussion

This study shows that, despite the presence of systemic inflammation, oral supplementation with EAAs can normalise serum albumin in the majority of hypoAlb patients and reverse anaemia in more than one third of subjects after HF.

### 4.1. Baseline Circulating Proteins

The hypoalbuminemia observed in both groups of patients at their admission to the Rehabilitation Institute is the result of several mechanisms including systemic inflammation (primed by both trauma and subsequent surgery) [40], the possible inadequacy of patients’ calorie-protein intake during their acute orthopaedic hospitalisation, alterations in body tissue composition, or poor nutritional status before the index event. 

In catabolic states such as trauma, surgery, and infection, Alb concentrations decrease by approximately 1–1.5 g/dL over a short time (3–7 days) [41]. This reduction in Alb is due to decreased synthesis, accelerated distribution from the intravascular space, and increased catabolism of the protein during metabolic stress [42]. During a catabolic period, low Alb may persist despite nutritional support and the exogenous administration of Alb [1]. 

Poor nutritional intake during an acute hospital stay is another factor that contributes to impaired circulating Alb levels because low calorie-protein intake reduces synthesis and accelerates the catabolism of Alb [5]. The patients in our study were likely to have had inadequate nutritional intake in the orthopaedic setting [29], as suggested by their low nutrition levels on entry to the Rehabilitation Institute The administered fluid contributed to lower serum albumin concentrations. We believe, however, that this was not important in the study patients as they were admitted to our Institute about 20 days from the index event, while the Extracellular Water ECW is usually lost several days after the acute event because of increased diuresis. In addition, no patient had clinical signs of water retention (edema), and all subjects were clinically and haemodynamically stable.

Both metabolic stress and inadequate nutrition can also explain the low levels of patient preAlb due to the fact that this negative protein of the acute phase response is sensitive to low nutritional intake [38].

The alteration of body tissue composition is another factor that could reduce Alb. Although body composition was not investigated in this study, the patients probably had reduced muscle mass following multiple catabolic factors such as metabolic stress, inflammation, and immobilisation. Even in relatively healthy, well-nourished elderly men and women, low serum Alb has been associated with reduced muscle mass [43].

Lastly, possible malnutrition at the time of fracture may have contributed to a low level of Alb in the patients in our study [44].

At admission to the Rehabilitation Institute, 67% of the entire patient population was anaemic. Reduced Hb levels in subjects with HF is a consequence of several factors including lower Hb on the day before fracture [19], bleeding and fluid shifts before surgery, a drop in Hb levels during surgery, and repeated phlebotomy [19]. Both preoperative Hb concentrations and perioperative bleeding are major determinants of anaemia in post-surgery HF patients. Perioperative transfusions, by inducing immune depression, [45] might have played an important role in favouring infectious complications in the study patients. In this way, transfusion may indirectly have contributed to reducing circulating protein levels. This suggests that it is important to increase presurgery Hb concentrations when needed in order to avoid blood transfusion [46]. Poor nutritional intake is also a co-factor of low Hb.

### 4.2. EAA-Associated Improvements in Alb and HB

This study provides a positive answer to our investigation into whether EAAs may increase concentrations of serum Alb and Hb. The results show that increases in Alb and Hb were similar in males and females. Indeed, at discharge, the percentage of hypoalbuminemic patients on EAAs dropped to about 31.7% from an initial 73.2%. The treatment group ingested more than double the amount of EAAs (from diet and supplementation) than the placebo group did. In addition, the contribution of EAAs to total amino acid content was higher in the treated group (95.5%) than in the placebo group (45%).

Given that both groups of patients had a similar intake of EAAs in their diet, the difference between the two groups in terms of albumin gain was clearly due to the EAA supplements that were given to the treated group.

Multiple mechanisms may explain the efficacy of EAAs in improving Alb and Hb. EAAs directly promote overall body protein synthesis [47] and inhibit proteolysis, which is particularly relevant to Alb [48]. These activities are present in several tissues including the liver, which is the site of Alb production [49]. Indirectly, EAAs stimulate body protein synthesis by increasing the biological activity of insulin-like growth factor-1 [50].

In addition, the leucine metabolite ß-hydroxy-ß-methylbutyrate (HMB) improves protein synthesis and reduces protein destruction, even in cancer subjects [51,52].

It is interesting to note that the amino acid tryptophan, contained in the mixture used in this study, can promote Alb production as it is the most important amino acid for Alb synthesis [53]. Indeed, in the liver, tryptophan stimulates the ribosomal re-aggregation leading to enhanced Alb production in a fasting state or in conditions of inadequate protein intake [54]. It is unlikely that diet played a role in improving Alb, given that nutritional intake was similar at admission to and discharge from our institute. It was also similar in both placebo and treated patients.

At discharge, patient body weight was similar to baseline values. This indicates that the ingestion of calories, even though it lower than recommended, met the patients’ actual body needs, suggesting that inactivity/immobilisation reduced their total body energy requirements.

The lower infection rate that occurred in EAA compared to placebo patients confirms the findings of our previous studies [35,54] and may be due to the fact that EAAs play an important role in improving immunological defences by inducing protein synthesis of immune cells [35]. The proimmunologic EAA activity may explain why infection did not preclude the improvements of Alb and Hb over time.

Lower infection rates probably aid Alb improvement. In this study, the improvement of Alb is in line with two investigations reporting EEA-induced Alb increase in sarcopenic patients with chronic obstructive pulmonary disease [32] and in institutionalized elderly subjects [33]. Conversely, our results are in contrast with a previous investigation, which showed that two-thirds of HF patients failed to increase their serum Alb despite both calorie and protein enrichment of a routine hospital diet [55]. This discrepancy may be reconciled considering the differences in the methodologies adopted. Indeed, ageing is associated with reduced anabolism efficiency in response to a normal protein meal [56], particularly under conditions of insulin resistance frequently found in post-traumatic elderly subjects. On the contrary, ingesting EAAs as free substrates can actually stimulate protein anabolism to a greater degree than amino acids from food proteins [57], even in diabetic subjects [58].

With regards to Hb, the positive influence of EAA supplementation was only partial, given that improvements in Hb, though significant, only occurred in just over a third of the anaemic patients. EAAs probably promote the synthesis rate of globin, the protein group of Hb. Considering the fact that globin consists of four polypeptide chains containing an extraordinarily high percentage of amino acids which are essential for maintaining its helicoidally form [59], supplemented EAAs can stimulate and enhance initiation, prolongation, and termination of the globin chain [59] involving RNA messengers, RNA transfers, and ribosomes [59]. It is interesting that over the first few months of the protocol for subjects on EAA, Hb improvement, unlike Alb, tended to be significant (*p* = 0.08). This difference could reflect the differences in the half-lives of the two proteins, i.e., 19–21 days for Alb and 7 days for Hb [59]. The positive correlation between Alb and Hb confirms the results of another previous study [22]. Notably, after EAA supplementation, this relationship was stronger than that observed under base conditions.

The study cannot explain why EAA failed to improve serum preAlb, which, like Alb, is a negative reactant of the acute phase response. At present, we can only postulate that inflammation may inhibit preAlb production more than Alb and/or that the synthesis of albumin was more sensitive than that of prealbumin to EAA activity.

The main finding of this study is the chance of improving the recovery of hypoalbuminemia and, to a lesser extent, of anaemia, despite the persistence of systemic inflammation. This should not be surprising, however, given that amino acid supplementation can be anabolic even during severe inflammation such as acute endotoxin-induced inflammation in humans [60]. Antinflammatory EAA activity may be partly due to hydroxy-methylbutyrate HMB, the efficacy of which was demonstrated in chronic obstructive pulmonary disease patients. CRP and white blood cells were shown to be significantly lower in patients in the treated group than in the control group [61].

The dose of supplemented EAAs may not have been sufficient for subjects who were still hypoalbuminemic (31.7%) or anemic (65.8%) at the end of the protocol. In addition, for these patients, the amino acid composition of the EAA mixture was not appropriate to exert a sufficient net synthetic activity, particularly when blood amino acid abnormalities coexisted.

Plasma amino acid alterations can be frequent following trauma/surgery or elective Hip arthroplasty [62]. The results of the current investigation are not in agreement with those found in gastric subjects on supplemented amino acids. However, the two studies are very different from both methodological and clinical context standpoints. Methodologically, gastric bypass subjects were provided with a mixture containing a high amount (24 g twice daily) of three amino acids only, not including EAAs, apart from a metabolite of leucine, as in our study. Arginine and glutamine may play a dual role in the intestinal tract, both protective and proinflammatory [63]. Nitric oxide (NO) overproduction from arginine supplementation has been related to greater colonic damage and inflammation [64]. Glutamine supplementation via the glutamic-citrulline-arginine metabolic pathway [65] may indirectly lead to NO formation. Interestingly, a diet providing 12% glutamine produces lower inflammation, and a diet containing 24% glutamine produces higher inflammation [66]. Supplemented EAAs in our patients did not produce inflammation but were compatible with a trend towards reduced CRP. From a clinical point of view, patients after gastric bypass surgery are in a condition of reduced alimentary intake, malabsorption, and a catabolic state. The patients in the present investigation were in a post acute phase of alimentary, functional, progressive recovery.

Our results found that, in the treated group, 31.7% of HF patients remained hypo-albuminemic and 65.8% remained anaemic at discharge from the Rehabilitation Institute. This raises the important issue of how to increase the number of patients with restored Alb and Hb levels.

The differences in Alb and Hb responses to EAA supplementation deserve to be mentioned. We believe that the factors that influence Alb concentrations, including the availability of tryptophan and methionine [50] (both contained in the formula used in the study), protein-energy intake [51], oncotic pressure [42], and hormones [42], may be easier to control than the factors that influence Hb synthesis and degradation, particularly in an inflammatory state such as relative or absolute erythropoietin (EPO) insufficiency [67] and bone marrow response to EPO. Indeed, inflammation may lower EPO levels and/or hamper the response to EPO [68]. Moreover, changes in the circulating levels of testosterone and thyroid hormones may render patients more susceptible to anaemia [69]. In brief, Hb synthesis may not solely depend on adequate provision of EAAs, but also on body status, EPO, and bone marrow response to EPO. For these reasons, we believe that restoring normal Hb levels in more than one third of the elderly patients with HF sequelae in our study by the simple supplementation of EAAs is clinically important.

## 5. Clinical Implications

oxygen The study suggests that it is beneficial to supplement EAAs to elderly patients with HF and concomitant mild hypoalbuminemia and/or anaemia. In this study, two months of EAA supplementation induced an average Alb increase of 0.22 g/dL, (+6%) compared to the baseline value. This change is quantitatively similar to that observed (+0.16 g/dL) in institutionalised elderly patients on an EAA mixture that was identical to the one that was used in the present study [33]. The importance of the degree of Alb improvement in this study may be highlighted by four considerations. Firstly, in elderly subjects, serum Alb levels >3.2 g/dL exert a protective effect on mortality 12 months after discharge from the Rehabilitation Institute [70]. Secondly, the physiological decrease in median Alb levels between ages 30 and 80 years is 9–12% for both men and women [22,71]. Thirdly, in clinical practice, the infusion of Alb in a chronic stable disease is an inefficient method to improve circulating protein because the exogenous supply of Alb increases degradation and reduces the synthesis rate of the protein [5]. Lastly, serum Alb is significantly associated with skeletal muscle mass [42], independently of age, dietary intake, frailty, physical activity, or morbidity. Improvement in Alb (and Hb) could reduce the risk of frailty in elderly subjects, particularly in those with skeletal trauma sequelae.

Although anaemia in elderly subjects after HF fracture does not seem to affect the risk of adverse outcomes at three, six, and 12 months after discharge from hospital [24], we believe that long-term improvement of low Hb levels may prevent patients from suffering from muscle alterations that cause an increased risk of frailty and falls [11,72]. Indeed, low Hb, by inducing chronic hypoxia [73] and higher levels of inflammatory markers [74], reduces muscle density, mass [11,75,76,77], strength [12], and microcirculation [78]. Muscle damage induced by low Hb is therefore in addition to the damage already produced by fracture, surgery, and immobilisation.

An important consideration for clinical practice is that the improvement of Alb and Hb levels in elderly subjects with low circulating proteins prevents decreased circulatory blood volume [79]. In turn, this causes the instability of arterial pressure and rheological alterations of circulation in vital organs.

It is interesting that our patients still had persistent inflammation 70–90 days after the acute event. This may have limited the number of patients who were able to benefit from EAA supplementation. Moreover, persistent inflammation suggests that CRP levels should be checked over time after patients are discharged, because persistent inflammation places subjects with sequelae of HF at an increased risk of delayed reacquisition of adequate motility and physical activity [35], progression of atherosclerosis, and proliferation of cardiovascular events [80]. Indeed, CRP may be involved in all stages of atherosclerosis by influencing processes such as the endothelial function, lipid effect, angiogenesis and apoptosis, thrombosis, complement activation, and monocyte recruitment and activation [81]. Elevated CRP may lead to the rupture of unstable arterial plaques, causing clinical manifestations of cardio-and/or cerebrovascular disease. Moreover, the persistence of inflammation may limit functional recovery after hip fracture surgery. Local and systemic inflammation, as indicated by increased CRP, favour muscle catabolism over anabolic activity. In our study, the Tumor Necrosis Factor (TNFα), which is the main proinflammatory cytokine, was not determined. TNFα induces a resistance to the growth hormone and reduces the levels of the potent anabolic IGF-1 [82]. Interleukin-6 (IL-6) is another important proinflammatory cytokine, which was not determined in this investigation. IL-6 stimulates liver production of CRP as well as hypothalamus-pituitary corticosurrenal axis [83] leading to cortisol overproduction that causes peripheral muscle insulin resistance and catabolic activity. Both TNF and IL-6, by mediating the inflammatory pathway, cause a shift in liver protein synthesis with increased acute phase protein and reduced non reactant proteins, among which is albumin. Thus, inflammation may contribute to patient muscle wasting, sarcopenia, and frailty, particularly when associated with malnutrition and vitamin D deficiency. This may help to explain why most re-admissions after HF surgery are for co-morbidity conditions such as infection or cardiovascular diseases and not for surgical complications [19]. This is relevant because impaired walking performance is permanent in 20% of HF patients [35,70], and there is a high institutionalisation rate (20–25%) [20]. This study points to a reconsideration of our hospital catering and patient education to a more healthy choice of food. Indeed the low amount of ω3 fatty acids ingested daily by the patients in this study could have favoured the persistent inflammation. Together with increased ω3 [84], vitamin E [85] and moderate alcohol consumption [86] have an impact on CRP levels. Thus, the intake of cold water, ocean fish, alcohol (red wine mainly 200 mL/d), and foods containing vitamin E such as nuts, pulses, grains, lentils, chickpeas and oats should be advised to inflamed HF patients.

## 6. Conclusions

This study indicates that oral supplementation of EAAs may enhance the recovery of hypoalbumenimia and anaemia in more than two thirds and one third, respectively, of inflamed elderly patients after HF surgery. The anabolic activity of EAAs occurs even in the presence of infection.

## 7. Limitations

This study has several limitations that need more specific research in order to be resolved. Circulating vitamin D levels were not evaluated. Normal vitamin D or its supplementation may be a factor that contributes to reducing inflammation both directly and indirectly. The vitamin directly regulates the immune system [87], thus playing an important role in patient susceptibility to hospital-acquired infections.

By reducing the risk of infection, vitamin D contributes indirectly to a reduced perpetuation of systemic inflammation [88]. A status of hypovitaminosis D is likely in the study patients. Firstly, the prevalence of suboptimal levels of 25-hydroxyvitamin D (25-OHD) has increased in the general population [89]. Secondly, inflammatory changes and intravenous fluid administration lead to a rapid drop (30–40%) in circulating vitamin D levels during acute stress [90]. In addition, inflammation is associated with a decreased vitamin D binding protein [91]. Thus, a vicious circle of vitamin D deficiency-inflammation might occur. Hypovitaminosis D may also reduce the positive effect of rehabilitation of patients with HF as vitamin D improves musculoskeletal function [92] and postural body sway [93] and reduces the number of falls [94]. Interestingly, a recent study has documented that, when sarcopenic elderly patients are supplemented with vitamin D, whey proteins, and essential amino acids, their physical activity decreases inflammation and increases fat-free mass and strength, functionality, and quality of life [95].

Measuring patient body composition and muscle strength would have strengthened the discussion regarding the improvement in circulating proteins and muscle mass/function [11,42]. An overall improvement of visceral and somatic proteins may be more important than the single factor to ensure that patients with HF have better body stability and performance of daily tasks. The quantification of ECW could indicate whether an excess of water retention still existed and could contribute to lower albumin concentrations [96].

It would have been useful to follow up discharged patients to document whether improved circulating Alb and, to a lesser extent Hb, could have actually influenced patients’ return to pre-fracture walking capacity [97], with the consequent effect of reducing the risk of physical frailty. Future studies will address HF patients suffering from co-morbidities that were initially excluded in the study (see the Section 2.1). Determining circulating levels of testosterone and thyroid hormones could contribute to finding the EAA mechanisms, which create improvement in proteins. These hormones are often altered in older individuals, and reduced levels render patients more susceptible to anaemia [26]. Knowledge of the blood amino acid profile may be useful to understand which is the best EAA formula composition for a particular patient.

In elderly subjects, the main nutritional factor for developing anaemia is obviously low iron availability, as indicated by low ferritin concentrations. Given that serum concentrations of this protein were not measured, it was not possible to exclude low iron availability as a nutritional factor contributing to anaemia. However, we believe that the persistent body inflammation observed in the study populations would have masked any possible hypoferritinemia from low iron intake.

As EAAs failed to normalise Alb in one third of patients and correct anaemia in about two thirds of patients, a well-planned study is needed to highlight the extent of the impact of infection on circulating proteins and whether this could be limited by increasing the amounts of supplemented EAAs and/or by changing the amino acid composition of the EAA formula.

Another limitation of the study was the fact that we only kept an alimentary diary for three days. We are aware that it would have been preferable to keep them for longer, although this would have been impractical for the nursing staff from an organisational point of view. However, in our Institute, the protocol for checking long-term patient food intake consists in keeping a daily qualitative (i.e., not weighing foods) diary and in frequently determining Blood Urea Nitrogen BUN to estimate the adequacy of protein (EAA) intake and creatinine levels.

## Figures and Tables

**Table 1:** Amino acid composition (mg) of a single packet (4 g) of treatment mixture (EAA group) or placebo mixture (casein group).

	EAA Group	Casein Group
	*Total amino acid (4 g) of which (mg)*	
Leucine	1250	380
Valine	625	272
Isoleucine	625	208
Lysine	650	308
Threonine	350	209
Cysteine	150	16
Histidine	150	104
Phenylalanine	100	192
Methionine	50	96.5
Tyrosine	30	209
Tryptophan	20	32
Serine	-	228
Proline	-	391.5
Glycine	-	52
Glutamic acid	-	801
Aspartic acid	-	268
Arginine	-	128
Alanine	-	105
EAA tot	3820	1801.5
% tot amino acids	95.5%	45%
BCAA	2500	860
% tot	62.5%	21.5%

**Table 2:** Demographic-, anthropometric-, co-morbidity index, biohumoral-, and nutritional- variables in two groups of patients after randomisation to either placebo or essential amino acid (EAAs) supplementation.

Variables	nv	Placebo Group (n°56)	EAA Supplemented Group (n°56)	*p* Value
**Demographic**				
Male/Female	-	27/29	25/31	0.3
Age (years)	-	81.4 ± 8.1	83.1 ± 7.5	0.15
**Anthropometric**				
Body weight (kg)	-	63.5 ± 18	62 ± 16.1	0.79
Body Mass Index (BMI) (kg/m^2^)	-	25.7 ± 7.9	24.9 ± 8.5	0.41
**Co-morbidity index (scores)**	-	1.8 ± 1.3	1.75 ± 1.2	0.78
**Biohumoral**				
Glucose (mg/dL)	78–110	98 ± 17	95 ± 8	0.8
Glycated hemoglobin (%)	≤6	6.3 ± 2.7	6.1 ± 1.8	0.71
Urea nitrogen (mg/dL)	4.67–23.3	24.6 ± 6	23 ± 9.1	0.69
Creatinine (mg/dL)	0.5–1.1	1.01 ± 0.6	1 ± 0.9	0.11
**Daily nutritional intake**	Recommended *			
Energy				
kcal	-	1511 ± 345	1460 ± 319	-
kcal/kg	29.4 M 27 F	23.8 ± 7.2	24.1 ± 6.4	0.9
Proteins				
g	-	58 ± 11	57 ± 13	-
g/kg	≥1.1	0.91 ± 0.2	0.92 ± 0.3	0.89
%Ė	-	15.3 ± 2.9	15.6 ± 3.5	-
*Providing EAAs (mg)*				
	Lysine	3810 ± 285	4093 ± 457	0.7
	Histidine	1669 ± 180	1624 ± 239	0.9
	Threonine	2362 ± 341	2258 ± 401	0.8
	Valine	3230 ± 454	3347 ± 398	0.8
	Isoleucine	2800 ± 375	2899 ± 315	0.9
	Leucine	4900 ± 615	4981 ± 585	0.9
	Methionine	1342 ± 302	1417 ± 412	0.7
	Phenyalanine	2600 ± 299	2757 ± 416	0.5
	Tryptophan	650 ± 72	690 ± 122	0.6
	Total	23,363 ± 2780	24,066 ± 2954	0.7
	% proteins	40.2 ± 4.8	42.2 ± 5.2	0.8
Carbohydrates				
g	-	171.5 ± 41	179.8 ± 51	-
g/kg	2.5–4	2.7 ± 0.55	2.9 ± 0.9	-
%Ė	-	45.4 ± 10.8	49.3 ± 14	0.78
Simple sugar				
g	-	64.4 ± 4.5	65.1 ± 3.2	-
%Ė	<15	17 ± 1.2	17.8 ± 0.9	0.9
Lipids				
g	-	66.3 ± 18	60.8 ± 16	-
g/kg	≤1	1.04 ± 0.4	0.98 ± 0.31	0.22
%Ė	<30	39.5 ± 2.76	40.1 ± 4.9	0.85
Saturated				
g		17.5 ± 3.9	12.1 ± 2.6	-
%Ė	<10	10.4 ± 2.5	7.45 ± 3.7	0.45
Monounsaturated				
g		40 ± 4.3	41.5 ± 6.8	-
%Ė		23.8 ± 2.5	25.6 ± 4.2	0.75
Polyunsaturated				
g		8.8 ± 2.9	7.2 ± 2.2	-
%Ė	5–10	5.2 ± 1.7	4.4 ± 1.34	0.8
Omega 6				
g		7.1 ± 2.8	6.1 ± 1.15	
%Ė	4–8	4.2 ± 0.45	3.76 ± 0.71	0.65
Omega 3				
g		1.7 ± 0.45	1.2 ± 0.6	
%Ė	0.5–2	0.01 ± 0.002	0.007 ± 0.003	0.81
Fibre (g)	>25	14.8 ± 4.3	21.7 ± 9.6	0.4
Calcium (mg)	1200 M; 1200 F	855 ± 184	786 ± 230	0.84
Phosphorous (mg)	700 M; 700 F	1050 ± 351	654 ± 251	0.2
Potassium (mg)	3900 M; 3900 F	2384 ± 146	2185 ± 192	0.85
Sodium (mg)	1200 M; 1200 F	1354 ± 139	1275 ± 235	0.78
Iron (mg)	10 M; 10 F	10.5 ± 3.7	9.8 ± 1.5	0.91
Zinc (mg)	12 M; 9 F	0.7 ± 0.15	0.95 ± 0.21	0.30
Thiamin (mg)	1.2 M; 1.1 F	1.1 ± 0.1	0.99 ± 0.14	0.9
Riboflavin (mg)	1.6 M; 1.3 F	1.25 ± 0.4	1.17 ± 0.15	0.75
Niacin (mg)	18 M; 18 F	14.7 ± 3.6	13.8 ± 2.5	0.85
Vitamin A (µg)	700 M; 600 F	585 ± 128	588 ± 97	0.97
Vitamin C (mg)	105 M; 85 F	75 ± 21	82 ± 32	0.88
Water (mL)	-	854 ± 160	794 ± 89	0.91

Data are expressed as mean ± standard deviation (SD); Statistical analysis: independent sample *t*-test and χ^2^-test for placebo group vs. EAA supplemented group; * Livelli di Assunzione di Riferimento di Nutrienti LARN 2014 [39].

**Table 3:** Changes over time of the study variables. T0 = baseline; T1 = 1 month; T2 = 2 months.

Circulating Proteins	Placebo *n* = 56			EAAs *n* = 56			*p* Interaction
	T0	T1	T2	T0	T1	T2	
Albumin g/dL (n.v. 3.5–5)	3.45 ± 0.34	3.50 ± 0.25	3.51 ± 0.34	3.47 ± 0.41	3.59 ± 0.48	3.7 ± 0.52	=0.038
Haemoglobin g/dL (n.v. ≥12 F; ≥13 M)	11.8 ± 1.7	11.7 ± 1.6	11.7 ± 1.6	11.4 ± 1.7	11.8 ± 1.7	12.2 ± 1.6	=0.008
Prealbumin mg/dL (n.v. 18–38)	15.9 ± 4	15.9 ± 3	16.1 ± 4.1	15.7 ± 5.7	18 ± 7.6	17.6 ± 6.1	=0.3
C-reactive protein mg/dL (n.v. <0.8)	9.3 ± 6.5	16.9 ± 16.1	10.1 ± 9.4	20 ± 17.8	24.5 ± 14.8	13.5 ± 9.3	=0.1
